# The Immunological Role of Vitamin D in Primary Immunodeficiencies: A Narrative Review of the Current Literature

**DOI:** 10.3390/biomedicines14020303

**Published:** 2026-01-29

**Authors:** Emanuela Zumbo, Federica Nuccio, Francesca Paladin, Giuseppe Murdaca, Sebastiano Gangemi

**Affiliations:** 1 School and Operative Unit of Allergy and Clinical Immunology, University Hospital of Messina, 98125 Messina, Italy; emanuela.zumbo.96@gmail.com (E.Z.); federica.nuccio01@gmail.com (F.N.); sebastiano.gangemi@unime.it (S.G.); 2 Elderly and Disabled Department, San Paolo Hospital, 17100 Savona, Italy; puell-a@hotmail.it; 3 Department of Internal Medicine, University of Genova, Viale Benedetto XV, 16132 Genova, Italy; 4 Allergology and Clinical Immunology Unit, San Bartolomeo Hospital, 19038 Sarzana, Italy

**Keywords:** vitamin D, calcitriol, VDR, primary immunodeficiency, autoimmunity, lymphocytes T, lymphocytes B, osteoporosis, artificial intelligence

## Abstract

Vitamin D is a fat-soluble hormone essential for bone mineralization. In addition to this role, vitamin D is known for its immunomodulatory effects through its binding to intracellular vitamin D receptors (VDRs), which translocate to the nucleus of immune cells, including antigen-presenting cells (APC), B lymphocytes, T lymphocytes and monocytes, thereby modulating the transcription of genes responsible for the immune response. Vitamin D deficiency is associated with an increased risk of developing infections and autoimmune diseases. The purpose of this review is to evaluate vitamin D deficiency in patients with primary immunodeficiency (CVID, XLA, DGS, APECED, SCID, WAS, HIES), to assess its clinical and therapeutic impact. Vitamin D deficiency, often asymptomatic, is associated with more severe clinical conditions in subjects with PID. It can be associated with osteoporosis, fractures, myelofibrosis and endocrine disorders such as hypocalcemia. These patients responded beneficially to calcitriol therapy, underscoring the need for long-term monitoring.

## 1. Introduction

Approximately 80% of vitamin D (VD) is produced in the body as cholecalciferol when we are exposed to sunlight, particularly UVB rays, which have a wavelength of 290–320 nm. The remaining 20% is obtained through food in the form of ergocalciferol from mushrooms or as cholecalciferol, primarily through the ingestion of milk, eggs, and fish.

25-hydroxylase converts ergocalciferol and cholecalciferol to calcidiol, which binds to VDPB and is transported to the kidneys. Alpha-1 hydroxylase converts calcidiol to calcitriol, the active form of VD, in the kidneys. Once it enters target cells, it acts as a lipid-soluble hormone and binds to the vitamin D receptor (VDR). The VDR–calcitriol complex in the cytosol is translocated to the nucleus, where it binds to the retinoid X receptor (RXR) to form a heterodimer, which interacts with the VD response element (VDRE) in vitamin D target genes [1].

VD performs several functions in our body, one of which, along with parathyroid hormone and calcitonin, is the regulation of calcium and phosphate homeostasis. The balance of these hormones is essential for proper bone mineralization [2,3].

VD also has an effect on the immune system, controlling the growth and differentiation of various cells. VDR is expressed in numerous cells of both the innate and adaptive immune systems (APCs, T lymphocytes, B lymphocytes, monocytes) [4,5].

**Figure 1:**
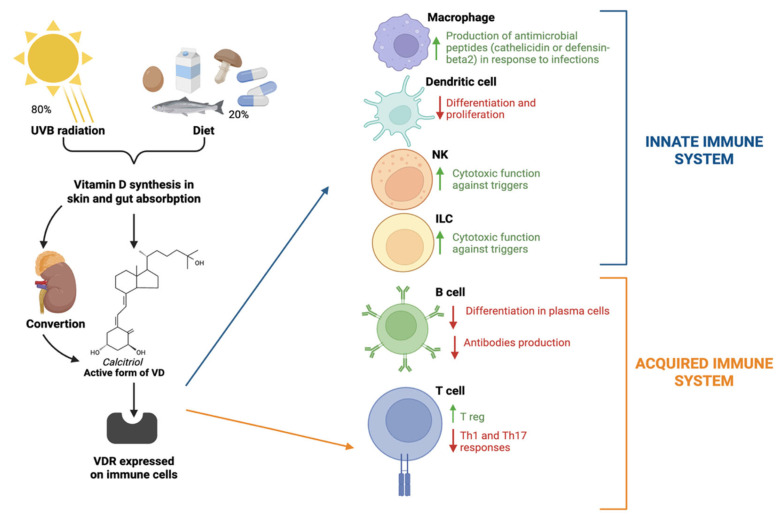
Schematic representation of Vitamin D metabolism and its immunomodulatory effects. Vitamin D synthesis and absorption mainly derive from UVB exposure and partially from external intake through diet or supplementation. After renal conversion to its active form, named calcitriol, it binds to the vitamin D receptor (VDR), an intracellular receptor expressed in immune cells that translocates to the nucleus and regulates the transcription of genes related to immune response with different subsequential immunological effects. Created in BioRender. Nuccio, F. (2025) https://BioRender.com/dggbncj.

Regarding the innate immune system, VD plays a role in the body’s defense against infections. In response to a bacterial infection or a pathogenic signal, macrophages and monocytes increase VDR expression. This upregulation occurs via the activation of Toll-like receptor (TLR) 1 and 2 expressed in immune cells, leading to VDR activation [6]. It enables the local conversion of 25(OH)D to active 1,25(OH)2D for autocrine/paracrine effects, including the production of antimicrobial peptides such as cathelicidin and defensin-beta2 [7]. On the other hand, it enhances the cytotoxic function of Natural killer (NK) and Innate Lymphoid Cells (ILCs) [8]. Furthermore, VD is also considered an immunomodulator, as it helps prevent autoimmune phenomena: it inhibits the differentiation of monocytes into dendritic cells, reduces the secretion of inflammatory cytokines (e.g., IL-6), and decreases Th1 and Th17-type immune responses, stimulating the production of regulatory T cells [9,10,11]. VD also exerts direct control over B lymphocytes by inhibiting their differentiation into antibody-secreting plasma cells and by suppressing B cell formation [12,13] (Figure 1).

Conversely, vitamin D deficiency favors a pro-inflammatory and autoimmune shift due to enhanced Th1 and Th17 responses, impaired Treg function and increased autoantibody production. These immunological effects provide a biologically plausible explanation for the evidence of an association between reduced circulating VD levels and the increased incidence and severity of autoimmune diseases, such as Multiple Sclerosis, Type 1 Diabetes, Psoriasis, Inflammatory Bowel Diseases, Rheumatoid Arthritis, Systemic Lupus Erythematosus, and many others [14,15].

Moreover, in the literature, several clinical trials have validated the therapeutic effects of VD and its metabolite supplementation in patients with autoimmune diseases [14,16]. Other factors, specifically gender and ageing, also correlate with reduced VD levels and changes in the gut microbiota, which can act synergistically and complementarily to promote a more inflammatory immune profile with effects on the susceptibility to and severity of immune diseases [17,18].

Immune dysregulation represents a common denominator between autoimmune diseases and several forms of primary immunodeficiencies (PIDs). Even if its role in these conditions as immunological diseases is not yet fully understood or explored, vitamin D may act as a critical immunological modifier.

As we know, patients with PID are at increased risk of infections as well as autoimmune diseases. Furthermore, some forms of PID, such as Hyper IgE Syndrome (HIES) due to a STAT3 defect, are associated with osteopenia and fractures [19], which are some of the possible complications influenced by altered calcium homeostasis. In fact, calcium–phosphate metabolism and bone remodeling are often impaired in PID affected population as a clinical consequence of a status of chronic inflammation, malabsorption, endocrine dysfunction and long-term therapies [20]. Current PID international diagnostic and management guidelines lack an explicit mention of routine vitamin D monitoring or supplementation as part of standard PID care [21]. Published practice parameters and position statements focus on immune-directed treatments like endovenous somministration of immunoglobulins, antibiotics, HSCT, and vaccines, but not on micronutrient supplementation, even if observational studies on nutritional deficiencies show that VD deficiency is common in the PID-affected population [22].

Given the increased burden of infections, autoimmunity and bone health in patients with inborn errors of immunity (IEI), the aim of this review is to characterize VD status in this population and to evaluate the immunological, clinical and therapeutic impact of its deficiency in disease outcomes and, therefore, to pave the way for future potential research areas.

## 2. Methods

The search was conducted on PubMed using the following MeSH terms: Vitamin D and Primary Immunodeficiency.

No lower date limits were set to include studies we deemed fundamental. The selected studies were those in English and ranged from 1994 to 2025. Of these, the most relevant studies that directly linked the role of vitamin D to the specific immunological defects described were selected. Of the 11 included articles, three were case reports, as these are very rare diseases and studies with large sample sizes have not yet been conducted.

## 3. Immunodeficiency B

B-cell or immunoglobulin immune disorders range from deficient B-cell development with complete absence of all Ig subclasses to deficiencies of a single Ig class or subclass [23,24]. Common Variable Immunodeficiency (CVID) is a primary immunodeficiency that represents the most common form in adults. It is characterized by reduced serum levels of IgG, IgA and/or IgM antibodies. This defect is linked to a failure of B lymphocytes to mature into antibody-secreting plasma cells [24].

Clinically, patients with CVID experience recurrent infections, especially respiratory (sinusitis, ear infections, bronchitis, pneumonia) and gastrointestinal infections. In more severe cases, patients may experience meningitis, osteomyelitis, sepsis, etc. Patients with CVID also have an increased risk of developing autoimmune diseases (immune thrombocytopenic purpura, hemolytic anemia, etc.), chronic inflammatory bowel diseases, and tumors, particularly non-Hodgkin’s lymphoma and stomach cancer [25].

Autoimmune endocrinopathies are another complication of CVID not only in adults, but also in pediatric ages, and include autoimmune thyroiditis, type 1 diabetes mellitus, vitamin D deficiency, and other complications [26].

Treatment is immunoglobulin (IgG) replacement therapy, which can be administered either intravenously or subcutaneously.

Although rare, familial forms of CVID exist, and the genes involved are ICOS, BAFFR, TACI and TNFRSF. In most cases, the cause is not yet known [27].

In 1999, an analysis was conducted to investigate the genetic basis of CVID clinical variability using the PCR-SSP (Polymerase Chain Reaction-Sequence Specific Priming) technique. This analysis showed that the granulomatous form was significantly associated with the rare TNF 488A allele and the IL-10 promoter haplotype a-t-a (associated with low IL-10 production). While alleles of VDR and interleukin-6 (IL-6) have been associated with immunophenotypic abnormalities characteristic of the more severe forms of the disease, the APAL polymorphism, a single-nucleotide genetic variation of a single nucleotide in the gene encoding VDR, has been analyzed. It is an intronic polymorphism and is associated with increased expression of VDR. In this case, the immunosuppressive effect of calcitriol is enhanced [28].

The role of the APAL polymorphism has subsequently been further evaluated in other autoimmune diseases. We report a comprehensive review in 2024 that found a strong association between this polymorphism and susceptibility to developing systemic lupus erythematosus, particularly in females [29].

Also in 2024, a study analyzed VDR genetic variants in blood samples from 206 patients with Crohn’s disease, highlighting that the VDR Apal Aa genotype and the VDR BsmI Bb genotype have a positive association with perianal involvement of the aforementioned disease [30].

Similarly, a 2018 meta-analysis of 17 studies reported that the APAL polymorphism is positively associated with an increased susceptibility to developing vitiligo [31].

VDR expression was analyzed in 2011 in three patients with CVID and asymptomatic VD deficiency, but in this study, VDR levels were found to be downregulated compared to healthy controls. Asymptomatic VD deficiency in patients with CVID, linked to a reduction in VDRs, increases the risk for these patients of bone complications, but also of infections, autoimmune diseases and neoplastic diseases, which are already known complications of CVID [32].

Osteoporosis, in fact, is a complication analyzed by Baris et al. as it represents a growing health problem in CVID. The risk of bone loss has been shown to increase with advancing age and the severity of lung damage (bronchiectasis) and to correlate with low levels not only of VD and calcium, but also of IgG and B lymphocytes [20].

In X-Linked Agammaglobulinemia (XLA), first described by Bruton in 1952, the primary defect is the inability of pre-B lymphocytes, normally present in the bone marrow, to mature into B lymphocytes. The genetic alteration underlying this pathology involves the BTK gene, present on the X chromosome, and, since the disorder is inherited recessively, mainly affects males, while females are more often carriers.

XLA is characterized by extremely low or absent serum levels of all immunoglobulin classes (IgG, IgA, IgM, and IgE), and the clinical manifestations appear early in childhood, once maternal antibodies have been lost. In this case, too, they are predominantly recurrent bacterial infections, such as sinusitis, otitis, pneumonia, and meningitis, and treatment consists of periodic infusions of Ig [33].

In 2017, a study comparing bone health in patients with Common Variable Immunodeficiency (CVID) and X-linked Agammaglobulinemia (XLA) was published.

A significant difference was observed between the two groups: 40.5% of CVID patients had low BMD compared to 15.8% of XLA patients. This study suggests that, in addition to factors common to both groups (nutritional, gastrointestinal, and infectious), CVID patients are more prone to bone marrow density (BMD) alterations due to complications specific to their condition, particularly lymphoproliferative disorders and endocrine disorders (autoimmunity) [33].

The association between VD and CVID is an increasingly important area of research that highlights a complex link between nutrition, bone metabolism, and immune function. Deficiency is often asymptomatic and therefore should be monitored to prevent or treat complications associated with CVID.

## 4. Immunodeficiency B and T

Combined immunodeficiency with B- and T-cell deficiencies refers to a significant deficiency of lymphocytes, which are essential for a proper immune response. Primary combined immunodeficiencies include Severe Combined Immunodeficiency (SCID), also known as “bubble baby disease.” SCID is not a single disease but a group of very rare, extremely severe inherited primary immunodeficiencies.

This deficiency or severe dysfunction of all the main components of the adaptive immune system results in an almost completely inactive immune system. Symptoms typically begin in the first months of life (between 3 and 6 months). Their condition is considered a medical emergency, as even mild infections, which are common in a healthy child, can be fatal in a child with SCID.

There are multiple forms of SCID, each caused by a specific genetic defect; for example, X-linked SCID associated with pathogenic variants in the common gamma chain of the receptors for IL-2, IL-4, IL-7, IL-9, and IL-15, and autosomal recessive SCID linked to genetic mutations in JAK-3. Other forms of SCID are linked to adenosine deaminase (ADA) deficiency, purine nucleoside phosphorylase deficiency, and recombinase 1 and 2 (RAG1/RAG2) deficiency.

In 1994, the first case was described of a child with SCID who developed myelofibrosis secondary to vitamin D-deficiency rickets. The patient also developed severe anemia and thrombocytopenia secondary to myelofibrosis.

Correction of VD deficiency in this patient had a significant clinical impact, resolving rickets and myelofibrosis and, consequently, restoring bone marrow function. However, this result should not be interpreted as definitive proof of efficacy, but suggests the importance of diagnosing and treating all comorbidities, including nutritional ones, in patients with immunodeficiencies [34].

Wiskott–Aldrich syndrome (WAS) is an X-linked disorder characterized by a triad of symptoms: bleeding diathesis, recurrent bacterial infections, and eczema. The gene involved is located on the X chromosome and encodes Wiskott–Aldrich Syndrome Protein (WASP), which is expressed by all hematopoietic stem cells. WASP is essential for the proper production, structure and release of platelets from the bone marrow, as well as for the migration, adhesion and cytoskeleton function of T and B lymphocytes.

Therefore, in this disease, B and T lymphocytes function abnormally, and T lymphocyte levels are decreased.

Even in this primary immunodeficiency, patients have an increased risk of developing autoimmune diseases (e.g., hemolytic anemia) and malignancies, particularly lymphomas, due to immune dysregulation.

A case report published in 2000 highlighted a new potential long-term complication of bone marrow transplantation (BMT) for WAS: severe osteopenia with bone fragility that responds favorably to calcitriol therapy, suggesting that assessment and management of bone health, particularly VD metabolism, should be an integral part of the long-term follow-up of patients undergoing BMT for primary immunodeficiencies such as WAS [35].

Hyper-IgE Syndrome (HIES) is a rare primary immunodeficiency disorder characterized by a distinctive triad of symptoms: elevated serum immunoglobulin E levels, recurrent infections, and cold abscesses.

There are two genetic variants: an autosomal dominant form resulting from alternations in the STAT3 gene, and an autosomal recessive form linked to a variant in the DOCK8 gene. There are even rarer forms in which the TYK2 and PDG genes are involved.

According to the updated classification of the International Union of Immunological Societies (IUIS), HIES falls primarily into the category of diseases involving defects of combined immunity and, more specifically, among defects in the function and number of T and/or B lymphocytes.

The most common form is AD-HIES. STAT3 is a transcription factor crucial for the response to many cytokines. A STAT3 pathogenic variant impairs the maturation of T-helper 17 lymphocytes, leading to dysregulation of B-cell differentiation. This results in excessive IgE production and a deficient antibody response.

This syndrome also involves non-immune organs, leading to physical and skeletal alterations such as scoliosis, osteopenia and increased fracture frequency.

The mechanisms underlying bone fragility in Autosomal Dominant Hyper-IgE Syndrome (AD-HIES) were investigated by Sowerwine et al., who highlight that the problem extends beyond simple reductions in bone mineral density (BMD). Despite evidence of increased osteoblastic activity, there are additional factors that influence frailty, and serum VD was not correlated with the number of fractures in this study [19]. The apparent contradiction may underscore the pathophysiological specificity of this syndrome. As we know, in HIES, the skeletal defect and high incidence of fracture are primarily related to the STAT3 defect, regardless of the VD axis.

## 5. Immunodeficiency T

Primary immunodeficiencies associated with T cell defects are characterized by impaired immune response, resulting in increased susceptibility to viral, fungal, and/or bacterial infections, as well as opportunistic infections. A key feature of these conditions is the altered expression of genes involved in immune regulation: reduced FOXP3 expression compromises the development and function of regulatory T cells (Tregs). This leads to a loss of suppressive activity against effector subpopulations, such as Th1 and Th17, resulting in dysregulation of immune homeostasis and an increased risk of autoimmune complications [36]. In addition, affected patients may have a combined defect (B and T cells) as the stimulatory action on antibody production is lost, which may be clinically indistinguishable from SCID.

DiGeorge syndrome (DGS), also known as chromosome 22q11.2 deletion syndrome (22q11.2DS), is a congenital disorder caused by a microdeletion on chromosome 22. This deletion results in an inadequate development of the third pharyngeal pouch during embryogenesis, from which the thymus originates, and the other derived anatomic structures, leading to consequent defective T-cell maturation and varying degrees of T-cell immunodeficiency, along with other physical abnormalities [37].

Indeed, clinical presentation is characterized by distinct craniofacial features and a highly variable phenotype. In most cases, a classic triad is recognized, including conotruncal cardiac anomalies, hypoplastic/aplastic thymus and underdevelopment of parathyroid glands, which are diagnosed early in life. The main consequences are immunodeficiency, whose severity varies with the degree of thymic hypoplasia leading to T-cell depletion, and hypocalcemia, respectively. Affected patients can experience recurrent infections and autoimmune or atopic disorders, which can affect the patient’s learning ability and psychiatric condition [38].

The entity and range of symptoms can vary widely among affected individuals and at different ages, and, in the absence of classic findings, a prompt diagnosis may be missed [39,40,41].

Although extremely uncommon—occurring in fewer than 1% of individuals with DGS—complete thymic aplasia is possible, and is linked to a severe combined immunodeficiency (SCID) phenotype [42].

Recent research highlights the multifaceted role of VD in DGS patients, especially related to immune system regulation and calcium metabolism.

As already mentioned, one of the possible clinical presentations of DGS patients is associated with immunity dysfunction leading to autoimmunity or poor defence against common and opportunistic infections. Patients with low VD levels exhibit significant reductions in naive T cells and plasmacytoid dendritic cells (pDCs), potentially contributing to their increased susceptibility to infections and risk of autoimmune diseases as they age. Moreover, VD could have an influence on thymic output as demonstrated by a direct link between its levels and recent thymic emigrant (RTE) cell numbers, newly produced T cells exiting thymus and entering peripheral circulation [43].

Hypocalcemia represents another potential life-threatening complication in DGS, varying from severe neonatal hypocalcemia to latent hypoparathyroidism [44].

Patients may present with jitteriness, tetany or seizures. Rarely, complications of hypocalcemia are the presenting symptoms of an undiagnosed DGS in adulthood [44,45,46].

VD deficiency can exacerbate hypocalcemia and has been identified as a risk factor specifically in patients with transient hypoparathyroidism, underscoring the need for careful assessment and management of VD status in these individuals. It is therefore recommended that adults with 22q11.2 deletion routinely receive calcium and vitamin D or its active form (calcitriol) supplementation to improve circulating calcium levels and to prevent complications arising from this metabolic condition [47].

Although causality remains difficult to prove in clinical settings, collectively these findings point to VD deficiency as a modifiable factor contributing to immune dysregulation and an increased risk of infections and autoimmunity in DiGeorge syndrome.

Autoimmune polyendocrinopathy–candidiasis–ectodermal dystrophy (APECED), also referred to as autoimmune polyendocrine syndrome type I (APS-I or APS1), is a rare monogenic autoimmune disorder caused by genetic mutations in the AIRE gene. In addition to the classic clinical triad including mucocutaneous candidiasis, hypoparathyroidism and Addison’s disease, affected patients may suffer from other endocrine and non-endocrine autoimmune diseases [48,49].

AIRE is a transcriptional factor expressed on medullary thymic epithelial cells (mTECs) that has a role in peripheral and central T cell tolerance by eliminating autoreactive T lymphocytes via negative selection [50,51]. A preclinical study using in vitro and ex vivo mouse models demonstrated that VD promotes AIRE function acting as a VDR co-activator, suggesting a direct role of VD signaling in the regulation of immune tolerance in thymic tissue. This interaction could be a plausible molecular explanation for the association between VD deficiency and AIRE-related immune dysregulation [52]. In fact, AIRE genetic defects lead to autoreactive T clones and consequent immune dysregulation. In these patients, immunodeficiency coexists with multiorgan autoimmunity. Impaired T cell tolerance is connected to B cell dysfunction and the subsequent aberrant production of high-affinity autoantibodies against different organs and cytokines responsible for the variable manifestations of this syndrome [53].

Autoantibodies against IL-6, IL-17, IL-22, IL-23, involved in anti-fungal immunity, have been detected in most APECED patients, thus explaining their higher susceptibility to infections and chronic mucocutaneous candidiasis (CMC) in particular.

The endocrine system, and the parathyroid gland in particular, is the most common target of tissue-specific autoimmunity [54].

The subsequent hypoparathyroidism may remain subclinical for a prolonged period; however, precipitating factors such as stress or infections can trigger potentially life-threatening hypocalcemic seizures or tetany.

Therefore, regular monitoring of serum calcium and phosphorus levels is recommended in all patients to prevent the acute onset of these complications [55].

Treatment with calcitriol or recombinant PTH is effective in achieving adequate calcium homeostasis; nevertheless, careful dose balancing is essential to minimize treatment-related effects, including hypercalciuria and nephrocalcinosis [56].

In this syndrome, VD expresses its functional duality: on the one hand it contributes to maintaining mineral balance compromised by hypoparathyroidism, on the other hand it influences the immune response altered by AIREgenetic alterations.

Although specific data on the role of VD in APECED are limited so far, evidence from other studies conducted on patients affected by other autoimmune polyglandular syndromes supports its potential immunomodulatory action. Specifically, Kraus et al. examined a cohort of APS-2 patients, demonstrating that the active form of vitamin D is able to modulate the inflammatory response of monocytes, reducing the expression of pro-inflammatory cytokines (IL-6, CCL-3, IL-23A) and thus increasing anti-inflammatory mediators such as IL-10. Moreover, a correlation with an HLA-DQ genetic background was demonstrated in these patients, with lower expression of VDR and other genes related to vitamin D in high-risk HLA-DQ variants [57].

As also shown by Bellastella et al., patients with other APSs have significantly reduced levels of 25-OH vitamin D compared to controls, regardless of the number of autoimmune disorders present. This evidence supports the possible pathogenetic role of this hormone in autoimmunity and as a therapeutic target also in APECED [58].

Table 1 illustrates the main findings of our literature research.

## 6. Highlights

- Asymptomatic hypovitaminosis D in CVID may increase the risk of developing complications, such as osteoporosis, infections, autoimmune diseases and neoplastic diseases (already known complications).
- VD is a modifiable factor that plays a multifactorial role in DiGeorge syndrome: therapeutic supplementation strategies should be considered to prevent both metabolic and autoimmune complications, such as hypocalcemia, and recurrent infections.
- In APECED, VD could represent a relevant therapeutic target having an influence both on mineral homeostasis induced by hypoparathyroidism and on immune dysregulation.
- A causal link between VD and combined immunodeficiencies has not been reported, but VD supplementation may help prevent/correct some complications of these pathologies.

## 7. Conclusions and Future Perspectives

In summary, our review highlights VD as a pleiotropic hormone that plays a crucial role in both metabolic homeostasis and immune regulation. VD deficiency is a common and often underdiagnosed condition in patients with primary immunodeficiencies (PIDs), including CVID, XLA, DiGeorge syndrome, APECED, SCID, WAS and others. These disorders are characterized by increased susceptibility to infections, immune dysregulation with autoimmune manifestations and significant endocrine and metabolic complications.

Across different forms of PID, including B- and/or T-cell defects, low vitamin D levels seem to not be strictly associated with more severe disease phenotypes and poorer clinical outcomes with higher risk of complications, but it is indirectly involved in immune regulation and prevention of autoimmune and metabolic complications typical of these rare conditions. Accordingly, several studies suggest that VD supplementation, either in its active form (calcitriol) or through recombinant PTH, may contribute to clinical improvement, especially in terms of bone health, while evidence on immunological or infectious outcomes remains limited. The immunomodulatory effect of VD is mediated by the activation of VDRs expressed in immune cells (APCs, T and B lymphocytes, monocytes) leading to enhanced antimicrobial peptide production, suppression of pro-inflammatory cytokines and promotion of regulatory T-cell function.

Despite these findings, causality is difficult to establish, as most available evidence is limited to small observational studies and case reports. Furthermore, the heterogeneity of PIDs in terms of genetic background, clinical course, disease severity and treatment strategies limits the possibility of drawing definitive conclusions because of possible disparate causal inferences.

Future research should focus on elucidating the immunomodulatory mechanism of VD in PIDs and on defining optimal, personalized supplementation strategies based on specific immunological, clinical and genetic profiles. Although current international guidelines do not specifically include recommendations for VD supplementation as a part of standard of care, routine monitoring of VD status and serum calcium levels, along with the prompt correction of deficiency, should be considered an integral component of the long-term management of patients with primary immunodeficiencies. However, evidence is currently insufficient to define PID-specific target VD levels or supplementation regimens.

## Figures and Tables

**Table 1:** Summary of the main findings of the studies taken into consideration in our review, highlighting the role of Vitamin D analyzed indicated with “Prognostic” if associated with disease severity, “Therapeutic” if its supplementation improves clinical outcomes and “Predictive” associated with increased immune or metabolic risk.

PID	Author, Year	Type of Study	Vitamin D Role	Main Findings	Clinical Relevance and Perspectives
CVID	Mullighan et al., 1999 [28]	Genetic association study	Prognostic	VDR gene polymorphism (APAL) correlates with severe clinical phenotypes; enhanced immunosuppressive effect of calcitriol	Genetic background influences disease severity and response to VD
	Ardeniz et al., 2008 [32]	Case series	Prognostic	Asymptomatic VD deficiency associated with downregulated VDR levels and higher risk of bone and other complications	VD levels should also be monitored in asymptomatic patients
	Baris et al., 2011 [20]	Observational study	Prognostic	Low VD and calcium levels correlated with the risk of osteoporosis and number of B and T lymphocytes	VD levels contribute to bone complications
CVID vs. XLA	Mohebbi et al., 2017 [33]	Comparative observational study	Prognostic	Reduced BMD more prevalent in CVID patients	VD monitoring particularly important in CVID patients
SCID	Al-Eissa et al., 1994 [34]	Case report	Therapeutic	VD deficiency rickets caused myelofibrosis and bone marrow dysfunction that ameliorated with correction of VD levels	VD supplementation can prevent multisystemic complications
WAS	Talkhani et al., 2000 [35]	Case report	Therapeutic	Severe osteopenia after BMT improved with calcitriol therapy	Long-term VD and bone monitoring required after BMT
HIES	Sowerwine et al., 2014 [19]	Cross-sectional study	Prognostic	Bone fragility not directly related to VD levels	Skeletal complications in AD-HIES are multifactorial
DiGeorge Syndrome	Legitimo et al., 2020 [43]	Observational study	Prognostic/Predictive	Low VD levels associated with reductions in naïve T cells, plasmacytoid dendritic cells and thymic output	VD may influence immune competence and infection risk
	Denkboy Öngen et al., 2023 [47]	Clinical cohort study	Therapeutic	VD supplementation improves calcium homeostasis and prevents hypocalcemia	VD or calcitriol supplementation is recommended to prevent complications
APECED (APS1)	Kraus et al., 2020 [57]	Ex vivo	Therapeutic	Therapy with calcitriol in APS2 patients reduced pro-inflammatory cytokines and increased IL-10 production; lower expression of VDR correlated with high-risk HLA-DQ variants	As in APS2, VD may modulate immune dysregulation in APS1; genetic background may have an influence on disease prognosis
	Bellastella et al., 2015 [58]	Observational study	Prognostic	Reduced 25-OH-D levels are frequent in other autoimmune polyendocrine syndromes	VD deficiency may also contribute to autoimmune burden in APECED

## Data Availability

No new data were created or analyzed in this study.

## Abbreviations

The following abbreviations are used in this manuscript:

VD	vitamin D
VDR(s)	vitamin D receptor(s)
APC(s)	antigen presenting cell(s)
PID	primary immunodeficiency
CVID	common variable immunodeficiency
APECED	Autoimmune Polyendocrinopathy–Candidiasis–Ectodermal Dystrophy
XLA	X-linked agammaglobulinemia
SCID	severe combined immunodeficiency
WAS	Wiskott–Aldrich Syndrome
DGS	DiGeorge syndrome
PTH	parathyroid hormone
